# Pregnancy incidence and associated risk factors of dichorionic triamniotic triplet under assisted reproduction: A large sample of clinical data analysis

**DOI:** 10.3389/fendo.2023.1049239

**Published:** 2023-03-17

**Authors:** Shuhua Liu, Qianhua Xu, Yafen Wang, Bing Song, Zhaolian Wei

**Affiliations:** ^1^Reproductive Medicine Center, Department of Obstetrics and Gynecology, The First Affiliated Hospital of Anhui Medical University, Hefei, China; ^2^Department of Obstetrics and Gynecology, Anhui Province Maternity and Child Health Hospital, Hefei, China; ^3^NHC Key Laboratory of Study on Abnormal Gametes and Reproductive Tract, Anhui Medical University, Hefei, China; ^4^Ministry of Education Key Laboratory of Population Health Across Life Cycle, Anhui Medical University, Hefei, China; ^5^Anhui Province Key Laboratory of Reproductive Health and Genetics, Hefei, China; ^6^First Clinical College, Anhui Medical University, Hefei, China

**Keywords:** dichorionic triamniotic, assisted reproductive technology, triplet pregnancies, incidence, risk factors

## Abstract

**Background:**

Dichorionic triamniotic (DCTA) triplet pregnancies are rare in spontaneous pregnancy. The aim was to characterize the incidence and risk factors of DCTA triplet pregnancies after assisted reproductive technology (ART).

**Methods:**

A retrospective analysis of 10,289 patients, including 3,429 fresh embryo transfer (ET) cycle and 6,860 frozen ET cycle, was performed from January 2015 to June 2020. The effect of different ART parameters on the incidence of DCTA triplet pregnancies was evaluated by multivariate logistic regression analyses.

**Results:**

Among all clinical pregnancies after ART, the incidence of DCTA was 1.24%. 1.22% occurred in the fresh ET cycle, while 1.25% occurred in the frozen ET cycle. The number of ET and cycle type has no effect on the occurrence of DCTA triplet pregnancies (*p* = 0.987; *p* = 0.056, respectively). There were significant differences in DCTA triplet pregnancies rate among receiving intracytoplasmic sperm injection (ICSI) and receiving *in vitro* fertilization (IVF) [1.92% vs. 1.02%, *p* < 0.001, *OR* = 0.461, 95% confidence interval (CI) 0.315–0.673], blastocyst transfer (BT) versus cleavage-ET (1.66% vs. 0.57%, *P* < 0.001, *OR* = 0.329, 95% CI 0.315–0.673), and maternal age ≥ 35 years versus maternal age < 35 years (1.00% vs. 1.30%, *P* = 0.040, *OR* = 1.773, 95% CI 1.025–3.066). Based on the regression analysis of cycle type, DCTA triplet pregnancies rate was higher in maternal age < 35 years than in maternal age ≥ 35 years (1.35% vs. 0.97%, *P* < 0.001, *OR* = 5.266, 95% CI 2.184–12.701), BT versus cleavage-ET (1.47% vs. 0.94%; *P =* 0.006, *OR* = 0.346, 95% CI 0.163–0.735), and receiving ICSI was higher than receiving IVF (3.82% vs. 0.78%, *p* < 0.001, *OR* = 0.085, 95% CI 0.039–0.189) in fresh ET cycle. However, DCTA triplet pregnancies rate did not show difference in maternal age, insemination methods, and number of ET, and only BT was found to be associated with a higher DCTA triplet pregnancies rate in the frozen ET cycle (1.73% vs. 0.30%, *p* < 0.001, *OR* = 0.179, 95% CI 0.083–0.389).

**Conclusion:**

The prevalence of DCTA triplet pregnancies has increased after ART. Maternal age < 35 years, BT, and receiving ICSI are risk factors for DCTA triplet pregnancies, also in fresh ET cycle. However, in frozen ET cycle, BT is an independent risk factor for increased DCTA triplet pregnancies rate.

## Introduction

Due to the widespread use of assisted reproductive technology (ART), the prevalence of multiple pregnancies has increased significantly over the past few decades (1, 2). Limiting the number of ET has a certain effect in reducing the incidence of multiple pregnancies during ART (3, 4). However, triplet and multiple pregnancies can also occur after single or double ET. Depending on the time of embryonic cell division, dizygotic triplets can be divided into triplet pregnancies including monochorionic monoamniotic (MCMA) twinning, trichorionic triamniotic (TCTA) triplet pregnancies, and dichorionic triamniotic (DCTA) triplet pregnancies according to chorionicity. A DCTA triplet pregnancy involves a monochorionic diamniotic (MCDA) twinning sharing a single placenta (5). MCDA pregnancies are associated with specific complications arising from common placental vascular anastomosis, such as twin anemia polycythemia sequence (TAPS), selective intrauterine growth restriction (SIGR), and twin-to-twin transfusion syndrome (TTTS), which affect infant and maternal morbidity and mortality (4, 6–8).

Our previous study showed that DCTA triplet pregnancies showed the worst pregnancy and obstetric outcomes (9). The exact reason for the increased incidence of DCTA triplet pregnancies after ART is unknown. Previous studies have shown that medium condition (10), maternal age (7, 11–13), micromanipulation of the zona pellucida (ZP) (11, 13–17), BT (14, 18–23), ovarian hyperstimulation (18), and genetics (24–27) may be factors contributing to the increased incidence of monozygotic twining (MZT) pregnancies. However, there is no definitive conclusion (17, 28–31). Our previous research showed that maternal age and BT are independent risk factors for increased monozygotic pregnancy rate and that BT in fresh cycle and maternal age in frozen cycle are the main factors contributing to MCDA twinning, respectively (32).

The mechanisms underlying the increased incidence of DCTA triplets after ART are rarely reported. Therefore, we retrospectively analyzed the clinical characteristics of embryonic and clinical pregnancy populations after ART to describe the incidence and risk factors of DCTA triplet pregnancies.

## Materials and methods

### Participants

We retrospectively analyzed all clinical pregnancies with fresh or frozen ET cycle performed at our center from January 2015 to July 2020, excluding donor oocyte cycles. All infertile patients underwent ovulation induction, follicle retrieval, conventional IVF or ICSI, and ET. All operations were performed by an experienced team of experts in our center, and informed consent of infertile couples was obtained. This project was supported by the Ethics Committee of the First Affiliated Hospital of Anhui Medical University.

### Interpretation of DCTA pregnancy results

In all patients conceived by ART, fetal heart beat status was identified by transvaginal ultrasonography in the first trimester (6–8 weeks) to determine chorionicity and fetal number. DCTA triplet pregnancies were confirmed when the fetal heart beat count is 3, and embryonic bud was observed in one of the gestational sacs.

### Statistical analysis

Maternal age, insemination methods (ICSI or IVF), culture length (cleavage or blastocyst stage), and number of ET are analyzed by SPSS 24.0 software package (SPSS Inc, Chicago, IL) between DCTA and non-DCTA groups. DCTA triplet pregnancy rates for each ET were assessed by multivariate regression analysis, and risk factors for DCTA triplet pregnancies in fresh and frozen ET cycles were analyzed. Risk factors were presented using forest plots GraphPad Prism version 9.0 for Windows (GraphPad Software, San Diego, CA, USA, www.graphpad.com). A *p*-value less than 0.05 was considered statistically significant.

## Results

### Probability of DCTA triplet pregnancies

**Figure 1:**
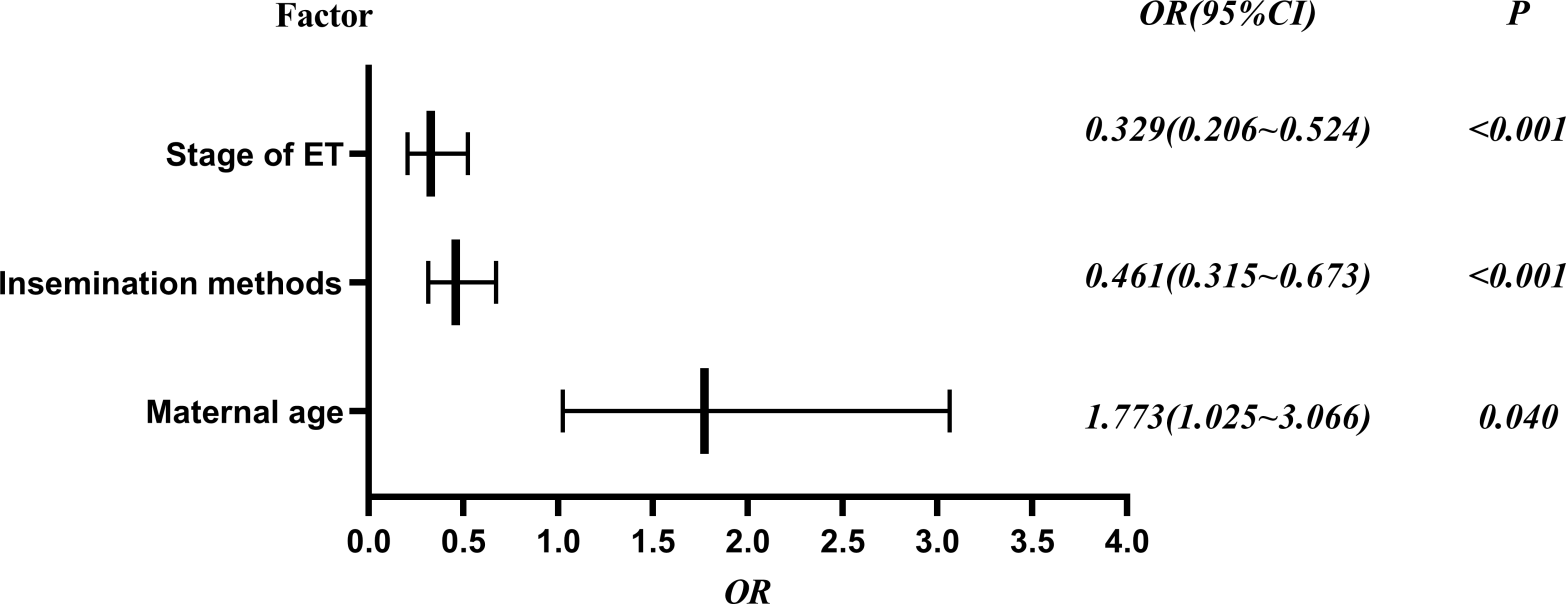
DCTA risk factor analysis forest plot, OR (95% confidence interval) and P values.

A total of 10,289 patients, including 3,429 from fresh ET cycle and 6,860 from frozen ET cycle, were conceived through ART from January 2015 to June 2020. Among all clinical pregnancies, the overall incidence of DCTA triplet pregnancies was 1.24% (128/10289). **Table 1** shows that, when classified by cycle type, the incidence of fresh ET cycle was 1.22% (42/3429) and the incidence of frozen ET cycle was 1.25% (86/6860). The number of ET was not a factor in the generation of DCTA triplet pregnancies (*p =* 0.987). Notably, receiving ICSI has a higher rate of DCTA triplet pregnancies than receiving IVF (1.92%, vs. 1.02%, *p* < 0.001). BT yielded a higher DCTA triplet pregnancies rate than cleavage-ET (1.66% vs. 0.57%, *P* < 0.001). Compared with maternal age ≥ 35 years, the incidence of DCTA was higher in maternal age < 35 years (*P* = 0.040). The OR values, 95% confidence intervals, and *P*-value of the associated risk factors causing the increase in DCTA triplet pregnancies are visually shown in **Figure 1**.

**Table 1:** Analysis of DCTA risk factors by multivariate logistic regression.

Factors	DCTA (n=128)	No-DCTA (n=10161)	Total	*P*
Cycle type				0.056
Fresh ET cycle (n,%)	42(1.22%)	3387(98.78%)	3429	
Frozen ET cycle (n,%)	86(1.25%)	6774(98.75%)	6860	
Maternal age(years) (n,%)				**0.040**
≧35 (n,%)	18(1.00%)	1784(99.00%)	1802	
<35 (n,%)	110(1.30%)	8377(98.70%)	8487	
Insemination methods				**<0.001**
ICSI (n,%)	49(1.92%)	2497(98.08%)	2546	
IVF (n,%)	79(1.02%)	7664(98.98%)	7743	
Number of ET				0.987
Single-ET (n,%)	0(0%)	1379(100%)	1379	
Multiple-ET (n,%)	128(1.44%)	8782(98.56%)	8910	
Stage of ET				**<0.001**
Cleavage-ET (n,%)	22(0.57%)	3867(99.43%)	3889	
BT (n,%)	106(1.66%)	6294(98.34%)	6400	

ET, embryo transfer; ICSI, intracytoplasmic sperm injection; IVF, *in vitro* fertilization; BT, blastocyst transfer.

Bold values was statistically significant (P<0.05).

### Risk factors for DCTA triplet pregnancies in fresh and frozen ET cycle groups

**Figure 2:**
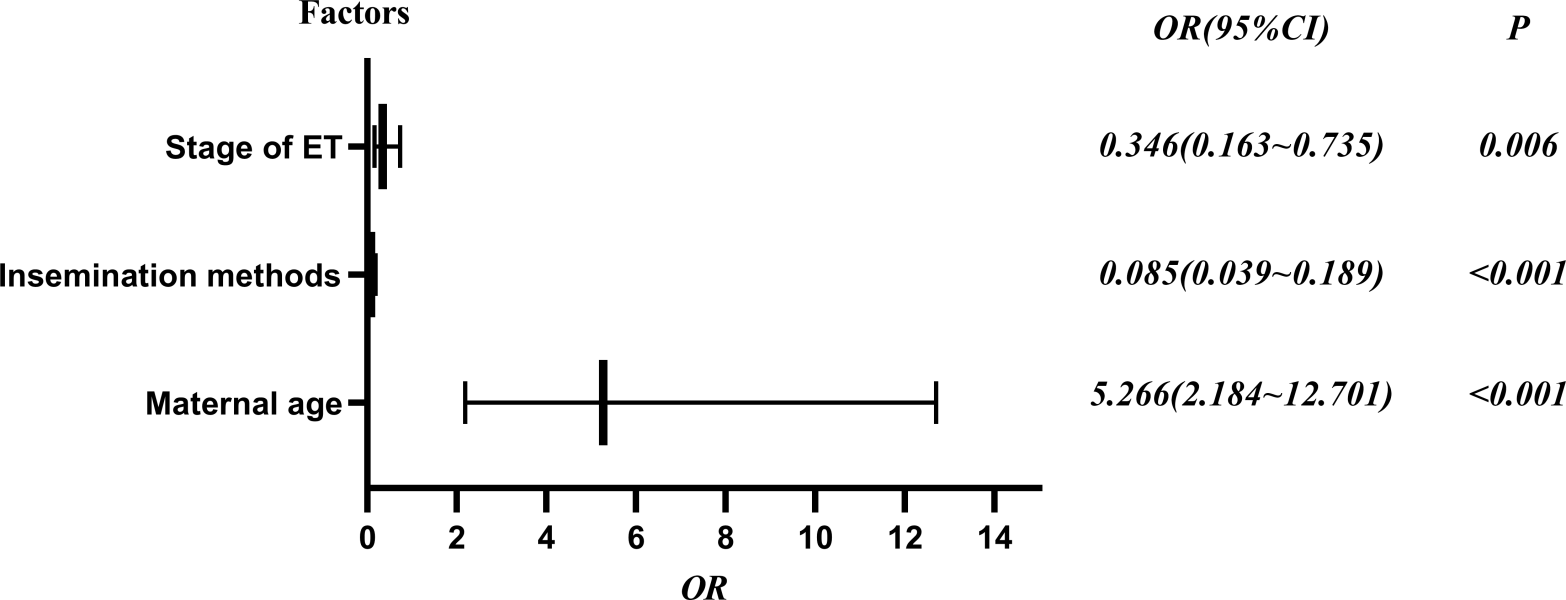
Risk factors of DCTA in the fresh ET cycle forest plot, OR (95% confidence interval) and P values.

**Figure 3:**
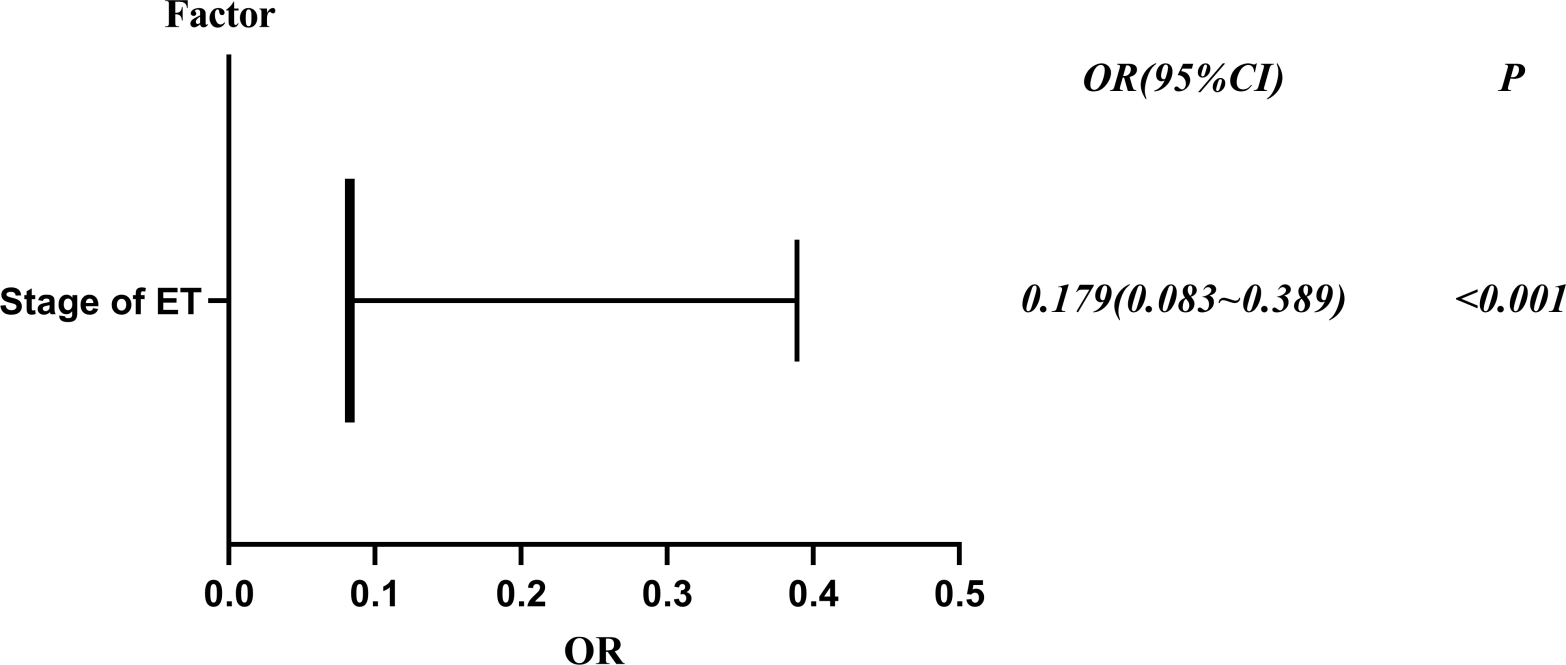
Risk factors of DCTA in the frozen ET cycle forest plot, OR (95% confidence interval) and P values.

To explore potential risk factors for DCTA triplet pregnancies, we conducted a series of related studies to determine the relationship between DCTA triplet pregnancies and ART-related procedures. According to cycle type, we divided the subjects into fresh ET cycle group and frozen ET cycle group. As shown in **Table 2**, the number of ET was not an influencing factor causing the increased rate of DCTA triplet pregnancies (*p* = 0.992); however, maternal age, insemination methods, and stage of ET were factors that cause the increased DCTA triplet pregnancies rate in the fresh ET cycle group. Maternal age < 35 years had a higher incidence of DCTA triplet pregnancies compared with maternal age ≥ 35 years (1.35% vs. 0.97%, *P* < 0.001) in the fresh ET cycle. BT was associated with an increased DCTA triplet pregnancies rate compared with cleavage-ET (1.47% vs. 0.94%; *P* = 0.006). Similarly, in **Table 3**, where we repeated the same analysis for the frozen ET cycle, DCTA triplet pregnancy rate was not associated with maternal age, insemination methods, stages, and number of ET. However, only BT was found to be associated with a higher DCTA triplet pregnancies rate (1.73% vs. 0.30%, *p* < 0.001). The OR values, 95% confidence intervals, and *P*-value of the associated risk factors leading to increased DCTA triplet pregnancies in the fresh ET cycle and frozen ET are shown in **Figures 2** and **3**, respectively.

**Table 2:** Risk factors of DCTA in the fresh ET cycle by multivariate logistic regression analyses.

Factors	DCTA (42)	No-DCTA (3387)	Total	*P*
Maternal age(years)				**<0.001**
≧35 (n,%)	11(0.97%)	1126(99.03%)	1137	
<35 (n,%)	31(1.35%)	2261(98.65%)	2292	
Insemination methods				**<0.001**
ICSI (n,%)	19(3.82%)	479(96.18%)	498	
IVF (n,%)	23(0.78%)	2908(99.22%)	2931	
Number of ET				0.992
Single-ET (n,%)	0(0%)	465(100%)	465	
Multiple-ET (n,%)	42(1.42%)	2922(98.58%)	2964	
Stage of ET				**0.006**
Cleavage-ET (n,%)	15(0.94%)	1575(99.06%)	1590	
BT (n,%)	27(1.47%)	1812(98.53%)	1839	

ET, embryo transfer; ICSI, intracytoplasmic sperm injection; IVF, *in vitro* fertilization; BT, blastocyst transfer.

Bold values was statistically significant (*P*<0.05).

**Table 3:** Risk factors of DCTA in the frozen ET cycle by multivariate logistic regression analyses.

Factors	DCTA (n=86)	No-DCTA (n=6774)	Total	*P*
Maternal age(years)				0.773
≧35 (n,%)	7(1.05%)	658(98.95%)	665	
<35 (n,%)	79(1.28%)	6116(98.72%)	6195	
Insemination methods				0.230
ICSI (n,%)	30(1.46%)	2018(98.54%)	2048	
IVF (n,%)	56(1.16%)	4756(98.84%)	4812	
Number of ET				0.990
Single-ET (n,%)	0(0%)	914(100%)	914	
Multiple-ET (n,%)	86(1.45%)	5860(98.55%)	5946	
Stage of ET				**<0.001**
Cleavage-ET (n,%)	7(0.30%)	2292(99.70%)	2299	
BT (n,%)	79(1.73%)	4482(98.27%)	4561	

ET, embryo transfer; ICSI, intracytoplasmic sperm injection; IVF, *in vitro* fertilization; BT, blastocyst transfer.

Bold values was statistically significant (*P*<0.05).

## Discussion

In China, we recommend that the number of transfer embryos should not exceed two Day 3 cleavage embryos or two blastocysts in the first cycle. The data show that, despite attempts to control the number of ET during ART, the triplet pregnancy rate remains above the spontaneous pregnancy rate of 0.03% (33). Multiple pregnancies have more maternal-fetal complications, especially involving a MCDA that shares common placenta and has vascular anastomosis (7). In our previous study, DCTA-expectant management had the worst pregnancy and obstetric outcomes compared with reduction to twin or singleton (9). In addition, we have reported factors associated with increased MCDA twinning after ART. Through a large cohort study, we found that the overall occurrence of MCDA twinning was approximately 2.55% of all clinical pregnancies after all ET cycles (32). This study found that the incidence of DCTA triplet pregnancies in all clinical pregnancies was close to 1.24% (128/10289). According to cycle type, the DCTA triplet pregnancies rate in fresh ET cycle and frozen ET cycle was 1.22% and 1.25%, respectively. Although DCTA triplet pregnancies contained MCDA twinning, our previous and this study found that there were significant differences between the incidence of DCTA triplet pregnancies and MCDA twinning. Furthermore, our two studies confirmed that the occurrence of DCTA triplet pregnancies and MCDA twinning was not associated with the number of ET. The difference in incidence may be related to the overall number of samples and clinical characteristics of different periods of the study subjects and may also be related to a more complex mechanism of DCTA triplet pregnancies than MCDA.

Studies have shown that the thickness of ZP decays with maternal age in the natural cycle, which may cause the embryo to be more susceptible to the division of the inner cell mass, and therefore older patients are more likely to have monozygotic twinning (7, 27). However, we found that maternal age < 35 years were more likely to develop DCTA triplet pregnancies than maternal age ≥ 35 years in fresh ET cycle after ART, suggesting that the mechanism behind the higher DCTA triplet pregnancies rate after ART differs from spontaneous pregnancies. The results of Liu X and his colleagues pointed out that there is an inverse relationship between the age of patients ≥ 36 years old and MZT, and patients younger than 36 years old are more likely to develop MZT (34). Likewise, investigators have seen a similar association between increased MZT and patients aged < 35 years in the donor oocyte cycle (7). This may be closely related to the high-quality embryos of young women, whose inner cell mass divides more easily.

We also found that ICSI was a risk factor for DCTA triplet pregnancies in fresh ET cycle but not in frozen ET cycle. This may be related to the fact that the ZP appears to be stiffer for frozen embryos than fresh embryos (35). Therefore, we guessed that ICSI may cause high-quality embryos with a weaker ZP more prone to DCTA triplet pregnancies in fresh ET cycle through this study.

A large number of studies have confirmed that BT is more prone to MZT (19, 20, 23, 36, 37). Similarly, our study also found that BT was also associated with the occurrence of DCTA triplet pregnancies, which is that BT had significantly higher DCTA triplet pregnancies than cleavage-ET (1.73% vs. 0.30%, *p* < 0.001) during frozen ET cycle. Currently, theories of possible mechanisms for how BT affects MZT rates have focused on changes in ZP and hatching process (38). However, some studies also have attributed the increased incidence of MZT to high-quality embryos (39) and high-grade trophectoderm (40). High-quality embryos may be mediated through human chorionic gonadotropin (hCG) secretion by more developed trophectoderms. In turn, increased hCG secretion may extend the implantation window to support embryonic division. Similarly, a study from a large sample data showed that the quality of the embryo cohort may be a key factor in the increased incidence of MZT after BT (39). We reasoned that high-quality blastocyst with strong developmental potential might be more likely to induce inner cell masses division during the frozen ET cycle, which suggests that ICSI and cryopreservation should not be listed as a risk factor for the increased incidence of DCTA triplet pregnancies in frozen ET cycle. It is also possible that the embryo has a rapid repair system when the ZP is damaged by ICSI (32).

Of course, our study will also have limitations. First, this study was inevitably limited by the fact that culture systems, ovulation induction procedure adopted, maternal antenatal risk factors, operating procedures changed during the study period. Unfortunately, some of the data collected came from imperfect clinical data, and some data were biased due to patient recall. These data can enrich our research through evaluation, and we hope to expand the sample data in future studies to supplement this part of the study. It is worth noting, however, that our clinical and laboratory procedures did not change significantly throughout the study to minimize possible confounding factors affecting the occurrence of DCTA triplets. Second, retrospective study is also a limitation of the study design, as study patients were not randomly assigned, which may lead to treatment and information bias. Given the relatively low incidence of DCTA triplet pregnancies, a prospective randomized controlled study is also inappropriate in this study. Our study was a single-center, large-sample cohort study, which excluded some effects of some changes in laboratory conditions and experimental operating procedures, which is actually the strengths of research design.

## Conclusions

After ART, the frequency of DCTA triplet pregnancies was significantly higher than that of spontaneous pregnancies. Maternal age < 35years, BT, and receiving ICSI are risk factors for DCTA triplet pregnancies, also in fresh ET cycle. However, BT is an independent risk factor for increased DCTA triplet pregnancy rate. Given the complications associated with DCTA triplet pregnancies, ART should be considered as a high incidence of DCTA triplet pregnancies and associated risk factor. Therefore, we need to consider and inform patients about the risk factors associated with the occurrence of DCTA triplet pregnancies in order to reduce the anxiety of patients and the associated risks of subsequent treatment when implementing ART.

## Data availability statement

The original contributions presented in the study are included in the article/supplementary material. Further inquiries can be directed to the corresponding authors.

## Ethics statement

The studies involving human participants were reviewed and approved by the Ethical Review Board of The First Affiliated Hospital of Anhui Medical University. The patients/participants provided their written informed consent to participate in this study. Written informed consent was obtained from the individual(s) for the publication of any potentially identifiable images or data included in this article.

## Author contributions

SL contributed to the conception and design. QX and BS contributed to the development of the methodology. SL collected and analyzed the data. SL, YW and BS contributed to the writing, review, and/or revision of the manuscript. BS and ZW contributed to administrative, technical, or material support. All authors contributed to the article and approved the submitted version.
